# Progressive sarcopenia and myosteatosis predict prognosis of advanced HCC patients treated with immune checkpoint inhibitors

**DOI:** 10.3389/fimmu.2024.1396927

**Published:** 2024-04-16

**Authors:** Mengchen Liu, Qianna Jin, Huiyan Wang, Yunqiao Li

**Affiliations:** ^1^ Department of Geriatrics, Union Hospital, Tongji Medical College, Huazhong University of Science and Technology, Wuhan, China; ^2^ Department of Radiology, Union Hospital, Tongji Medical College, Huazhong University of Science and Technology, Wuhan, China; ^3^ Hubei Province Key Laboratory of Molecular Imaging, Wuhan, China

**Keywords:** sarcopenia, myosteatosis, immunotherapy, advanced hepatocellular carcinoma, prognosis

## Abstract

**Background:**

Immunotherapy stands as a pivotal modality in the therapeutic landscape for the treatment of advanced hepatocellular carcinoma, yet responses vary among patients. This study delves into the potential impact of sarcopenia, myosteatosis and adiposity indicators, as well as their changes during immunotherapy, on treatment response and prognosis in patients with advanced hepatocellular carcinoma treated with immune checkpoint inhibitors.

**Methods:**

In this retrospective analysis, 116 patients with advanced hepatocellular carcinoma receiving immune checkpoint inhibitors were recruited. Skeletal muscle, intramuscular, subcutaneous, and visceral adipose tissue were assessed by computed tomography at the level of the third lumbar vertebrae before and after 3 months of treatment. Sarcopenia and myosteatosis were evaluated by skeletal muscle index and mean muscle density using predefined threshold values. Patients were stratified based on specific baseline values or median values, along with alterations observed during the treatment course. Overall survival (OS) and progression-free survival (PFS) were compared using the log-rank test and a multifactorial Cox proportional risk model.

**Results:**

A total of 116 patients were recruited and divided into two cohorts, 81 patients for the training set and 35 patients for the validating set. In the overall cohort, progressive sarcopenia (*P*=0.021) and progressive myosteatosis (*P*=0.001) were associated with objective response rates, whereas progressive myosteatosis *(P*<0.001) was associated with disease control rates. In the training set, baseline sarcopenia, myosteatosis, and subcutaneous and visceral adipose tissue were not significantly associated with PFS and OS. In multivariate analysis adjusting for sex, age, and other factors, progressive sarcopenia(*P*=0.002) and myosteatosis (*P*=0.018) remained independent predictors of PFS. Progressive sarcopenia (*P*=0.005), performance status (*P*=0.006) and visceral adipose tissue index (*P*=0.001) were all independent predictors of OS. The predictive models developed in the training set also had good feasibility in the validating set.

**Conclusion:**

Progressive sarcopenia and myosteatosis are predictors of poor clinical outcomes in patients with advanced hepatocellular carcinoma receiving immune checkpoint inhibitors, and high baseline visceral adiposity is associated with a poorer survival.

## 1 Introduction

Hepatocellular carcinoma (HCC) stands as a prevalent malignant neoplasm globally, ranking as the fourth leading cause of cancer-related mortality worldwide (1). Systemic therapies, including immunotherapy, are the mainstay of treatment for advanced HCC. Therapy with immune checkpoint inhibitors (ICIs) such as targeting programmed cell death 1 (PD-1) and programmed cell death ligand 1 (PD-L1) has been proven to be effective anti-tumor agents in HCC (2). Although checkpoint immunotherapy has changed the treatment paradigm for HCC, not all patients appear to respond to it. Hence, the major unresolved challenges of checkpoint immunotherapy for HCC are the discovery and validating of objective and measurable predictive biomarkers, identifying patients who will benefit from ICIs and advancing treatment to earlier stages of the disease.

Sarcopenia is a condition characterized by decreased skeletal muscle mass and muscle deterioration (3). During recent years, there has been growing evidence that sarcopenia was common in patients with a variety of cancers and was associated with a poor prognosis (4). A meta-analysis has reported that patients with sarcopenia have poorer survival and response to immunotherapy in comparison to those without sarcopenia, suggesting a negative correlation between sarcopenia and survival in cancer patients (5).

Furthermore, muscle depletion could be accompanied by an increase in the proportion of intermuscular and intramuscular fat. Excessive fat deposition in muscle as a result of abnormal distribution of adipose tissue in skeletal muscle is referred to as myosteatosis (6). Previous studies have demonstrated that myosteatosis was a poor negative indicator of cancer treatment response and prognosis (7, 8).

Additionally, the prognostic value of adiposity in various malignancies remains controversial. Most of the previous relevant studies used body mass index to assess the degree of adiposity, and a study argued that high body mass index (BMI) patients with metastatic cancer receiving immunotherapy had longer overall survival (9). However, the role of adipose composition (including visceral and subcutaneous adipose) on the survival of patients with advanced cancer is not known.

Overall, research on the impact of sarcopenia and myosteatosis on advanced HCC patients treated with ICIs is scarce. In addition, the role of changes in skeletal muscle and intramuscular adiposity as prognostic markers in HCC patients treated with ICIs has not been investigated to date. Therefore, the aim of this study was to evaluate the relationship and predictive significance of sarcopenia, myosteatosis, visceral adiposity, subcutaneous adiposity, and changes throughout the course of treatment in patients with advanced HCC treated with ICIs in relation to treatment response and prognosis.

## 2 Materials and methods

### 2.1 Patients

572 patients who were treated with ICIs immunotherapy for advanced HCC between April 2020 and March 2022 were consecutively enrolled in the survey. Criteria of included subjects:(1) patients aged 18 years; (2) patients categorized as Child-Pugh Class A or B; (3) patients treated with ICIs immunotherapy for advanced HCC; and (4) patients evaluated by CT before treatment within 1 month. Criteria of Excluded subjects:(1) patients receiving less than three courses of ICIs immunotherapy; (2) patients categorized as Child-Pugh Class C; (3) patients without CT at 3 months after treatment; and (4) patients with only MRI evaluation. A total of 116 cases were included in the final data analysis. The follow-up of all patients continued until 31 March 2023. This retrospective observational study was approved by the Medical Ethics Committee of Union Hospital, Tongji Medical College, Huazhong University of Science and Technology (reference number 0284) and waived the requirement for informed consent as all data were analyzed retrospectively and anonymously. And this study was conducted in accordance with the ethical principles of the Declaration of Helsinki and STROBE guidelines (10).

### 2.2 Treatment and tumor response assessments

ICIs immunotherapy, incorporating PD-1 inhibitors (camrelizumab, sintilimab, pembrolizumab, tislelizumab) and PD-L1 inhibitor (atezolizumab), includes combination therapies with anti-angiogenic agents. Tumor responses, assessed using RECIST 1.1 about three months post-treatment, categorize outcomes as complete remission (CR), partial remission (PR), stable disease (SD), or progressive disease (PD) (11). Key metrics include the objective response rate (ORR), representing CR and PR, and the disease control rate (DCR), encompassing CR, PR, and SD.

### 2.3 Image analysis

An experienced radiologist performed a CT scan evaluation of the patients using a SOMATOM Definition AS+ scanner (Siemens Healthcare, Erlangen, Germany) within 1 month before and 3 months after immunotherapy. Muscle and fat analysis utilized SliceOmatic version 5.0 software (TomoVision). Specific Hounsfield unit (HU) thresholds were applied to quantify skeletal muscle, subcutaneous adipose tissue (SAT), and visceral adipose tissue (VAT). Skeletal muscle was quantified by a Hounsfield unit (HU) threshold of -29 to +150. SAT was measured in the range of -190 to -30 HU and VAT in the range of -150 to -50 HU. Skeletal muscle, VAT, SAT, and VAT/SAT indices were calculated by normalizing the area (cm^2^) derived from the third lumbar (L3) vertebra to height squared (m^2^). Assessment of intramuscular fat levels by CT was mainly based on mean muscle density (MMD, measured in HU) at the level of the L3 vertebrae since the muscle radiodensity decreases with intramuscular adipose deposition (6).

According to the Japanese Society of Hepatology guidelines for sarcopenia in patients with liver disease, sarcopenia was defined as L3 skeletal muscle index (SMI)≤38 cm^2^/m^2^ in women and L3SMI ≤ 42 cm^2^/m^2^ in men (12). Myosteatosis was defined as MMD<41 HU in patients with a body mass index (BMI)<25 kg/m^2^, and MMD<33 HU in patients with a BMI≥25 kg/m^2^, on the basis of previous studies (13). Due to the lack of a standard cut-off value for adiposity measurements, the median values of the VAT, SAT, and VAT/SAT indexes were used to categorize the patients. Relative changes (%) throughout treatment were calculated as $\Delta x=\frac{index\;at\;3\;months-baseline\;index}{baseline\text{ ​}index}*100\%$ with a threshold defined as 10%. Progressive sarcopenia was defined as a >10% reduction in ΔSMI and progressive myosteatosis was defined as a >10% reduction in ΔMMD.

### 2.4 Statistical analysis

Statistical analysis and figure productions were conducted using SPSS version 26.0 (IBM) and RStudio version 2023.09.0 + 463. The continuous variables were displayed as mean ± standard deviation (SD) with categorical variables presented as totals and percentages (%). Comparison of continuous variables was performed by t-test or Mann-Whitney U-test, whereas categorical variables were analyzed by chi-square test or Fisher’s exact test. OS was defined as the time between first ICIs administration and death. PFS was defined as the time from first ICIs treatment to progression or death from any cause. PFS and OS were calculated using the Kaplan-Meyer method and relevant variables were analyzed using the log-rank test. Meaningful predictors were derived using LASSO regression in order to avoid the multicollinearity issue and to filter the variables at the same time. The univariate and multivariate Cox proportional hazards regression models were used to estimate predictors of PFS and OS. Statistical significance was set at *P*< 0.05.

## 3 Results

### 3.1 Patient characteristics

Between April 2020 and March 2022, 572 patients were initially enrolled, with 456 excluded for various reasons. The final analysis included 116 patients, randomized into a training set (81 patients) and a validating set (35 patients). Patient characteristics are detailed in **Table 1**, with a mean age of 53.96 years, mean BMI of 22.54 kg/m^2^, and a predominantly male cohort (77.6%). Most had hepatitis B virus infection (77.6%), ECOG performance status grade 0 (75.9%), Child-Pugh Class A (84.5%), and were in BCLC stage C (56.9%) at immunotherapy initiation. About 51.7% had prior hepatic partial hepatectomy. The most common immunotherapy agents were camrelizumab (41.1%), sintilimab (24.1%), and atezolizumab (12.1%). Baseline and 3-month values for skeletal muscle mass, intramuscular adipose tissue, subcutaneous adipose tissue, and visceral adipose tissue are in **Table 2**. The median SAT, VAT and VAT/SAT ratio indexes were 34.27 cm^2^/m^2^, 31.83 cm^2^/m^2^ and 0.945, respectively. Radiological evaluation showed CR in 4 patients, PR in 31, SD in 38, and PD in 43. Objective tumor response was 30.2%, and disease control rate was 62.9%. The median follow-up period of the total cohort was 20.4 months (95% confidence interval (CI):17.2-23.1 months). The median PFS and OS were 8.4 months (95% CI:6.0-10.3) and 15.8 months (95% CI:12.6-23.7), respectively.

**Table 1:** The characteristics of the enrolled patients.

Variables	Overall	Training set	Validating set
Number	116	81	35
Gender (Male), n	90(77.6%)	64(79.0%)	26(74.3%)
Age (year), mean ± SD	53.96 ± 12.16	54.23 ± 11.55	53.31 ± 13.63
BMI (kg/m^2^), mean ± SD	22.54 ± 2.70	22.37 ± 2.46	22.93 ± 3.19
ECOG PS, n			
0	88(75.9%)	61(75.3%)	27(77.1%)
1	28(24.1%)	20(24.7%)	8 (22.9%)
Child–Pugh Class			
A	98(84.5%)	66(81.5%)	32(91.4%)
B	18(15.5%)	15(18.5%)	3(8.6%)
BCLC stage			
B	50(43.1%)	31(31.3%)	19(54.3%)
C	66(56.9%)	50(61.7%)	16(45.7%)
PD-1/L1			
Camrelizumab	48(41.4%)	33(40.7%)	15(42.9%)
Sintilimab	28(24.1%)	21 (25.9)	7(20.0%)
Atezolizumab	14(12.1%)	8 (9.9%)	6(17.1%)
Tislelizumab	11(9.5%)	7 (8.6%)	4(11.4%)
Other	15(12.9%	12(14.8%)	3(8.6%)
PD-1/L1 combined with other therapies	51(44.0%)	40(49.4%)	11(31.4%)
Previous treatment			
Hepatic partial hepatectomy	60 (51.7%)	40 (49.4%)	20 (57.1%)
TACE	43 (37.1%)	30 (37.0%)	13 (37.1%)
Radiotherapy	17 (14.7%)	11 (13.6%)	6 (17.1%)
Hepatitis B virus infection	90 (77.6%)	61 (75.3%)	29 (82.9%)
Portal vein thrombosis	42 (36.2%)	34 (42.0%)	8 (22.9%)
Extrahepatic metastasis			
Lung metastases	16 (13.8%)	11 (13.6%)	5(14.3%)
Abdominal Metastases	26 (22.4%)	20 (24.7%)	6(17.1%)
Bone metastases	15 (12.9%)	10 (12.3%)	5(14.3%)
CRP≥8(mg/dL)	83(71.6%)	56(69.1%)	5(14.3%)
AFP≥ 400(ng/mL)	17(14.7%)	13(16.0%)	4 (11.4%)
Tumor response			
Complete remission	4 (3.4%)	1(1.2%)	3(8.6%)
Partial remission	31(26.7%)	20(24.7%)	11(31.4%)
Stable disease	38(32.8%)	33 (40.7%)	5(14.3%)
Progressive disease	43(37.1%)	27(33.3%)	16(45.7%)
Objective response rate	35(30.2%)	21 (25.9%)	14(40.0%)
Disease control rate	7 (62.9%)	54 (66.7%)	19(54.3%)

**Table 2:** The body composition of the enrolled patients.

Variables	Overall	Training set	Validating set
SMI (cm^2^/m^2^), mean±SD			
baseline	46.18±7.39	46.55±7.13	45.34±7.98
at 3 months	44.09±6.90	44.07±6.64	44.12±7.57
MMD(HU), mean±SD			
baseline	38.21±7.14	38.09±6.94	38.51±7.66
at 3 months	36.35±6.83	36.37±6.91	36.30±6.72
VAT index(cm^2^/m^2^), mean±SD			
baseline	34.86±22.69	34.19±22.42	36.39±23.56
at 3 months	36.42±24.17	35.31±23.14	38.96±26.58
SAT index(cm^2^/m^2^), mean±SD			
baseline	33.95±18.94	33.39±19.60	35.24±17.54
at 3 months	37.36±20.05	37.05±21.03	38.09±17.83
VAT/SAT ratio, mean±SD			
baseline	1.02±0.54	1.03±0.56	1.00±0.50
at 3 months	1.10±0.55	1.13±0.59	1.03±0.42

### 3.2 Relationship between body composition and treatment response

**Figure 1:**
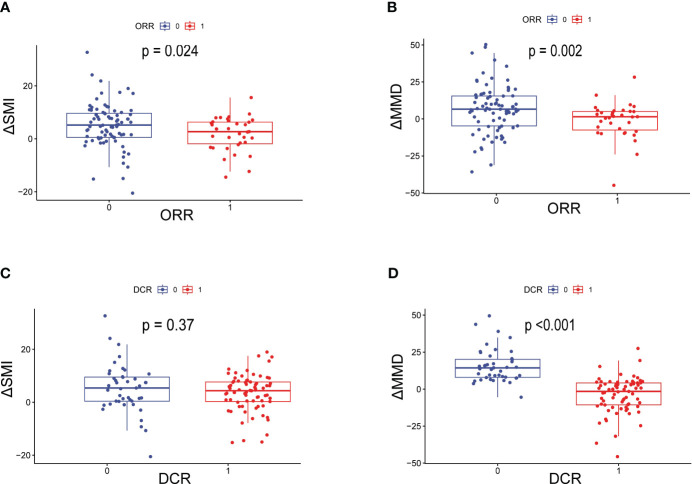
Differences in ΔSMI and ΔMMD levels in patients with or without ORR or DCR. **(A)** Difference in ΔSMI and **(B)** ΔMMD in patients with or without ORR; **(C)** Difference in ΔSMI and **(D)** ΔMMD in patients with or without DCR. ORR=objective response rate; DCR, disease control rate; ΔSMI, rate of change in skeletal muscle mass index; ΔMMD, rate of change in mean skeletal muscle density.

The data related to baseline body composition and post-treatment changes were first analyzed to compare differences in body composition in patients with or without ORR or DCR. There were no significant differences in baseline SMI, MMD, visceral fat content, or subcutaneous fat content in patients with or without a treatment response. As shown in **Figure 1**, patients without ORR had significantly greater changes in SMI (*P*=0.024) and MMD (*P*=0.002) compared with patients with ORR treatment response. Similarly, the change in MMD (*P*<0.001) was more pronounced in patients without DCR treatment response. Then, comparisons were carried out between different body composition subgroups in relation to treatment response. As shown in **Table 3**, progressive sarcopenia (*P*=0.021) and progressive myosteatosis (*P*=0.001) were associated with ORR, whereas progressive myosteatosis (*P*<0.001) was associated with DCR. Overall, patients with progressive sarcopenia or progressive myosteatosis had a poor response to treatment.

**Table 3:** Relationship between body composition indicators of patients and response to treatment.

Body composition	ORR			DCR		
	No	Yes	P	No	Yes	P
Sarcopenia			0.98			0.341
No	62 (76.5%)	26 (74.3%)		30 (69.8%)	58 (79.5%)	
Yes	19 (23.5%)	9 (25.7%)		13 (30.2%)	15 (20.5%)	
Myosteatosis			0.492			0.093
No	42 (51.9%)	15 (42.9%)		26 (60.5%)	31 (42.5%)	
Yes	39 (48.1%)	20 (57.1%)		17 (39.5%)	42 (57.5%)	
VAT index (cm^2^/m^2^)			0.053			0.701
≤ 31.83	35 (43.2%)	23 (65.7%)		20 (46.5%)	38 (52.1%)	
> 31.83	46 (56.8%)	12 (34.3%)		23 (53.5%)	35 (47.9%)	
SAT index(cm^2^/m^2^)			1			0.502
≤ 34.27	41 (50.6%)	17 (48.6%)		27(62.8%)	30(60.0%)	
>34.27	40 (49.4%)	18 (51.4%)		16 (37.2%)	20 (40.0%)	
VAT/SAT ratio			1			1
≤ 0.945	41(50.6%)	17 (48.6%)		22 (51.2%)	36 (49.3%)	
>0.945	40 (49.4%)	18 (51.4%)		21 (48.8%)	37 (50.7%)	
Progressive sarcopenia			0.021			0.202
No	63 (77.8%)	34 (97.1%)		33 (76.7%)	64 (87.7%)	
Yes	18 (22.2%)	1 (2.9%)		10 (23.3%)	9 (12.3%)	
Progressive myosteatosis			0.002			<0.001
No	53 (65.4%)	33 (94.3%)		17 (39.5%)	69 (94.5%)	
Yes	28 (34.6%)	2 (5.7%)		26 (60.5%)	4 (5.5%)	
ΔVAT index >10%			0.504			0.059
No	51(63.0%)	25 (71.4%)		23 (53.5%)	53 (72.6%)	
Yes	30 (37.0%)	10 (28.6%)		20 (46.5%)	20 (27.4%)	
ΔSAT index >10%			1			0.775
No	64 (79.0%)	28 (80.0%)		33 (76.7%)	59 (80.8%)	
Yes	17 (21.0%)	7 (20.0%)		10 (23.3%)	14 (19.2%)	
ΔVAT/SAT >10%			0.169			0.15
No	38 (46.9%)	22 (62.9%)		18 (41.9%)	42 (57.5%)	
Yes	43 (53.1%)	13 (37.1%)		25 (58.1%)	31(42.5%)	

### 3.3 Variables related to PFS and OS in the training set

**Figure 2:**
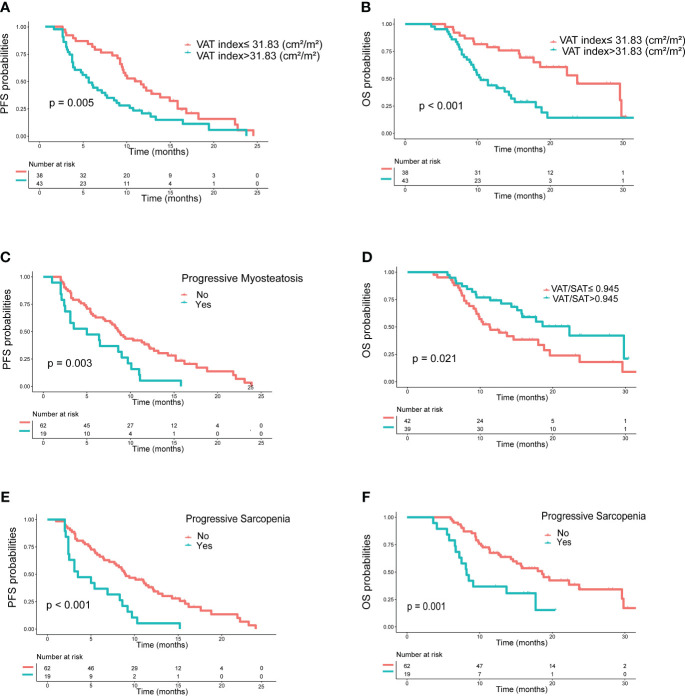
Kaplan−Meier curves of progression-free survival (PFS) and overall survival (OS) according to body compositions. **(A)** PFS and **(B)** OS based on the difference in VAT index; **(C)** PFS based on the presence or absence of progressive myosteatosis; **(D)** OS based on the difference in VAT/SAT; **(E)** PFS and **(F)** OS based on the presence or absence of progressive sarcopenia. P values were calculated using the log-rank test. VAT, visceral adipose tissue; SAT, subcutaneous adipose tissue.

In the training set, as shown in **Table 4**, clinical variables and baseline body composition as well as changes that may have an impact on prognosis were stratified and their effects on PFS and OS were explored using Kaplan-Meier analysis. Among the body composition indicators, baseline sarcopenia, myosteatosis, SAT index, and post-treatment changes in SAT and VAT index were not associated with PFS and OS. Kaplan-Meier curves showed (**Figure 2**) that patients with VAT index>31.83 cm^2^/m^2^ had significantly worse PFS (median, 5.0 vs. 10.6, *P*=0.005) and OS (median, 10.4 vs. 23.7, *P*=0.002) compared with patients with baseline VAT index ≤ 31.83 cm^2^/m^2^. Meanwhile, baseline VAT/SAT ratio was associated with OS (*P*=0.021) and progressive myosteatosis was associated with PFS (*P*=0.003). Patients with progressive sarcopenia had shorter PFS (median, 3.5 vs. 8.9, *P*<0.001) and OS (median, 8.2 vs. 18.6, *P*=0.001) compared with those without progressive sarcopenia.

**Table 4:** Kaplan-Meier analysis of factors associated with PFS and OS.

Variables	PFS		OS	
	Median time 95% CI (months)	*P*	Median time 95% CI (months)	*P*
Age (year)		0.391		0.747
<45	8.3(2.8-13.2)		16.8(7.8-20.6)	
45-59	8.7(6.4-12.1)		18.0(11.4-24.8)	
>60	5.9(3.5-15.8)		11.3(8.9-18.6)	
Gender		0.438		0.07
Female	5.5 (3.4-11.1)		10.4(9.5-9.6)	
Male	8.5 (6.5-10.3)		17.7(13.7-20.1)	
ECOG PS		0.002		0.001
0	8.8(6.9-11.3)		19.6(14.3-22.3)	
1	6.0(3.2-12.7)		8.2(6.9-14.6)	
Child–Pugh Class		0.020		0.02
A	8.7(6.4-11.1)		18.0(13.7-29.8)	
B	6.0(3.1-9.2)		8.5(7.2-13.6)	
BCLC stage		0.154		0.03
B	9.4 (5.7-12.1)		19.6(14.3-28.8)	
C	7.8 (4.7-10.1)		13.7(9.5-18.6)	
Portal vein thrombosis	6.0(3.1-9.2)	0.234	13.7(9.5-18.3)	0.167
Extrahepatic metastasis	7.4 (5.4-10.1)	0.558	9.4(7.8-13.4)	0.019
AFP ≥ 400 (ng/mL)	7.9(2.3-12.8)	0.618	15.1 (7.5-21.7)	0.649
CRP ≥8(mg/L)	8.2(3.6-10.9)	0.232	9.5(6.2-17.8)	0.007
Sarcopenia	5.2(3.2-12.1)	0.264	10.3(7.8-18.9)	0.324
Myosteatosis	8.3(5.4-12.1)	0.548	15.1(12.6-23.7)	0.789
VAT index>31.83 (cm^2^/m^2^)	5.0(3.2-8.2)	0.006	10.4(9.1-15.1)	0.002
SAT index>34.27 (cm^2^/m^2^)	6.3(4.1-9.2)	0.338	14.3(9.4-23.1)	0.287
VAT/SAT ratio ≤ 0.945	6.0(4.1-10.1)	0.337	11.3(9.5-19.6)	0.021
Progressive sarcopenia	3.5(2.5-9.2)	<0.001	8.2 (7.2-11.2)	0.001
Progressive myosteatosis	5.0 (3.1-10.1)	0.003	10.4 (7.5-15.6)	0.436
ΔVAT index>10%	6.9(3.4-14.3)	0.942	15.1(9.5-19.6)	0.800
ΔSAT index >10%	7.8(5.4-9.4)	0.39	14.6(11.3-22.3)	0.387
ΔVAT/SAT ratio>10%	6.5(3.4-10.3)	0.129	13.7(9.5-24.7)	0.16

**Figure 3:**
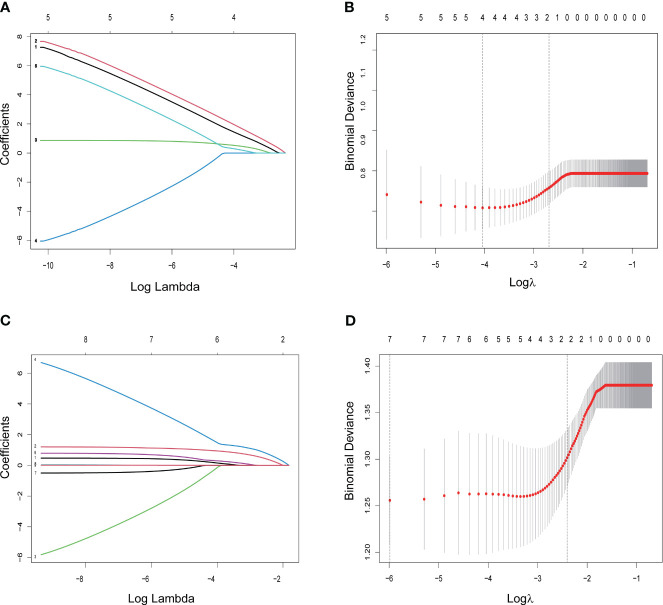
LASSO regression for screening predictors. **(A)** Distribution of LASSO coefficients for 5 factors associated with progression-free survival (PFS); **(B)** LASSO regression analysis identifying 2 factors for PFS; **(C)** distribution of LASSO coefficients for 8 factors associated with overall survival (OS); **(D)** LASSO regression analysis identifying 3 variables associated with OS.

An initial analysis of five variables related to PFS and eight variables related to OS were included. Based on LASSO regression analysis to filter variables, progressive sarcopenia and progressive myosteatosis were screened as predictor variables of PFS. ECOG PS, VAT index and progressive sarcopenia were screened as predictive variables for OS (**Figure 3**). The above filtered factors were subjected to multifactorial COX regression analysis to remove confounding factors. As shown in **Table 5**, both progressive sarcopenia (Hazard Ratio, HR=2.446, *P*=0.002) and progressive myosteatosis (HR=1.965, *P*=0.018) were independent predictors of PFS. Progressive sarcopenia (HR=2.525, *P*=0.005), ECOG PS (HR=2.365, *P*=0.006) and VAT index (HR=2.713, *P*=0.001) were independent predictors of OS.

**Table 5:** Multifactorial COX regression analysis of factors associated with PFS and OS.

Variables	PFS	*P*	OS	*P*
	HR (95% CI)		HR (95% CI)	
ECOG PS				
0	/	/	Ref.	Ref.
1	/	/	2.365 (1.280-4.371)	0.006
VAT index>31.83 (cm^2^/m^2^)	/	/	2.713 (1.482-4.967)	0.001
Progressive sarcopenia	2.446(1.387-4.321)	0.002	2.525 (1.317-4.844)	0.005
Progressive myosteatosis	1.965(1.122-3.441)	0.018		

### 3.4 Establishment and validating of predictive models

**Figure 4:**
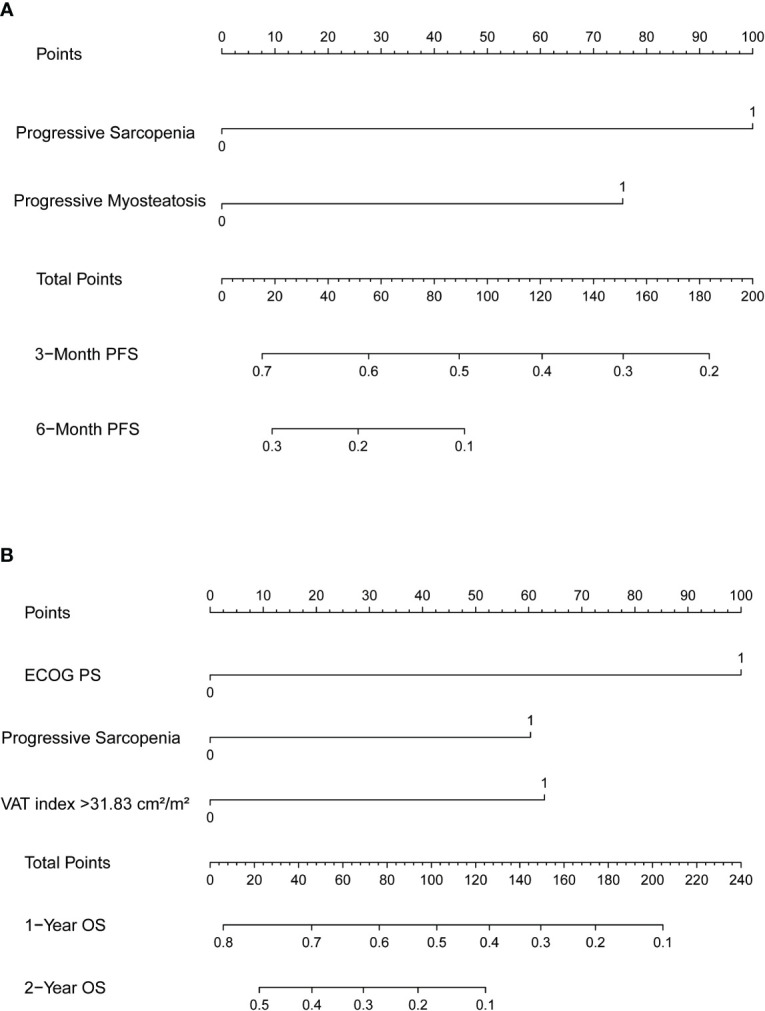
Nomograms predicting **(A)** 3-month and 6-month progression-free survival (PFS) and **(B)** 1-year and 2-year overall survival (OS). SMI, skeletal muscle index; MMD, mean muscle density.

**Figure 5:**
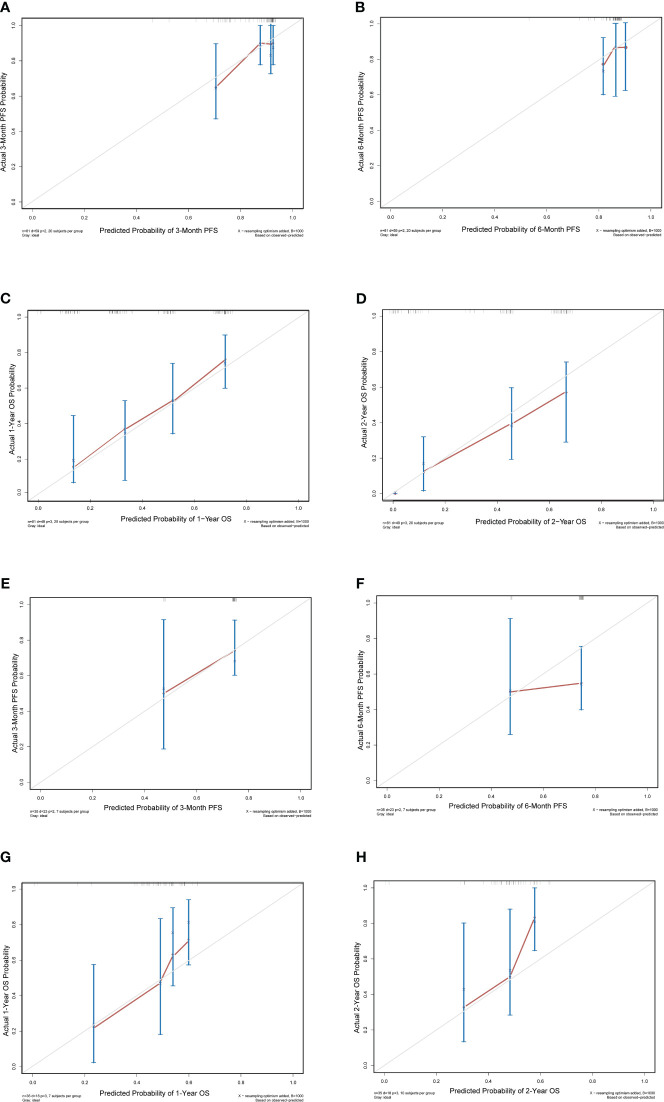
Calibration curves for progression-free survival (PFS) and overall survival (OS) in the training set and validating set. **(A)** 3-month PFS; **(B)** 6-month PFS; **(C)** 1-year OS and **(D)** 2-year OS in the training set. **(E)** 3-month PFS; **(F)** 6-month PFS; **(G)** 1-year OS and **(H)** 1-year OS in the validating set.

Nomograms predicting PFS at 3 and 6 months and OS at 1 and 2 years were established (**Figure 4**). The C-indices for the prediction models of PFS and OS were determined to be 0.706 (95% CI: 0.616-0.796) and 0.734 (95% CI: 0.671-0.808), respectively, indicating good discriminatory capability. Then, further validating of the predictive model was performed by using calibration curves and internal validating. The calibration curves for PFS (at 3 and 6 months, **Figure 5**) and OS (at 1 and 2 years) demonstrated that the predicted probabilities of PFS and OS closely aligned with the actual observed outcomes. In terms of external validating, the C-indices for the PFS and OS prediction models in the validating set were 0.694 (95% CI: 0.627-0.759) and 0.748 (95% CI: 0.685-0.811), respectively, with calibration curves also demonstrating good feasibility of the prediction models.

## 4 Discussion

In this study, we examined the ramifications of CT-based evaluation of dynamic changes about body components, encompassing skeletal muscle mass and adipose tissue during the course of treatment. Our objective was to elucidate the influence of these factors on tumor response and prognosis among patients with advanced HCC undergoing ICIs therapy. It was found that baseline sarcopenia, myosteatosis and subcutaneous adiposity tissues were not associated with immunotherapy outcomes. Notably, progressive sarcopenia and progressive myosteatosis were significantly related to adverse outcomes in patients with advanced HCC treated with ICIs during the course of treatment. To our knowledge, this is the first report to assess in detail by CT the clinical impact of body composition and its changes throughout the course of treatment in patients with advanced HCC treated with ICIs, and the first to demonstrate that high visceral adiposity is associated with adverse OS.

While numerous studies have investigated the impact of sarcopenia on survival in cancer patients, its prognostic significance in HCC patients undergoing immunotherapy remains a subject of debate. Moreover, the association between adiposity and immunotherapy outcomes in cancer patients is yet to be conclusively established. A prior study by Chen et al. demonstrated an association between sarcopenia and adverse outcomes in individuals with HCC undergoing treatment with ICIs (14). Nevertheless, Matsumoto et al. examined the influence of changes in muscle volume on outcomes in patients with unresectable HCC receiving atezolizumab plus bevacizumab (15). According to their results, a reduction in SMI was significantly associated with PFS, whereas the presence of sarcopenia did not exhibit a clear association with PFS.

Baseline sarcopenia and myosteatosis were detected to be irrelevant to PFS and OS in our study, in contrast to the findings of Chen et al. This may be a result of differences in patient selection, inconsistency in the discriminatory criteria, and so on. A uniform criterion for the demarcation of sarcopenia and myosteatosis is currently lacking, with large disparity between different races and patients. Therefore, we additionally assessed the levels of muscle and intramuscular fat three months into the treatment, conducting respective comparisons to eliminate confounding factors. It was observed that progressive sarcopenia was correlated with poor PFS and OS as well as treatment response, and that progressive myosteatosis was correlated with poorer PFS and treatment response.

It has been reported that muscle loss is highly prevalent in cancer patients, ranging from 10% to 90% (16). Skeletal muscle plays a pivotal role in modulating the tumor microenvironment and influencing immunomodulation through the secretion of a variety of myokines, such as Irisin, IL-15. Irisin has been reported to exert potential anticancer activity by disrupting the tumor-promoting microenvironment by inducing white adipose tissue browning and inhibiting macrophage infiltration (17). Research conducted by Milos has demonstrated that IL-15 may promote tumor immunity by inducing circulating CD8 T-cells and NK-cells (18). Reduced muscle quality will produce less IL-15, affecting the development and survival of natural killer cells, and may ultimately mediate tumor cell immune escape (19). In conclusion, the loss of skeletal muscle may contribute to a decrease in actin secretion, promoting tumor growth and suppressing tumor immunity, thereby influencing patient survival.

Furthermore, CT was utilized to enhance the accuracy of adiposity-related assessments. Interestingly progressive myosteatosis was discovered to associated with worse immunotherapy response and PFS, whereas high baseline visceral adiposity was associated with OS. On the one hand, progressive intramuscular adipose tissue deposition represents poorer muscle quality. Multiple interactions exist between skeletal muscle tissue and the immune system (20). Skeletal muscle cells could express major histocompatibility complex molecules to deliver antigens to T cells. Poorer muscle quality may mediate the immune tolerance of tumor cells to ICIs by affecting T cell function (21). On the other hand, as the amount of adipose tissue increases, the amount of proinflammatory factors such as leptin, IL-1 and IL-6 increases (22). Wang et al. found that leptin induced T-cell dysfunction in obese patients (23). In summary, progressive myosteatosis may significantly reduce the efficacy of ICIs. Additionally, high visceral adiposity may result in the accumulation of lipid intermediates, impair insulin signaling pathways, and induce insulin resistance, fostering an environment conducive to tumor growth (24), which in turn affects the survival of patients. Thus, fat accumulation in both skeletal muscle and visceral organs are not beneficial to immunotherapy and prognosis in patients with hepatocellular carcinoma, and more research is needed in the future to focus on and validate the effect of fat distribution on tumors. In addition to this, the prediction model incorporated the ECOG PS clinical score, which has been shown to be an important predictor of prognosis in immunotherapy (25).

These findings underscore the significance of ongoing monitoring of changes in body composition, with a specific emphasis on muscle and intramuscular fat, within the realm of clinical practice. Rather than exclusively focusing on pre-treatment baseline metrics, oncologists and radiologists should remain vigilant to alterations over the course of treatment. This proactive approach enables timely adjustments or the incorporation of interventions into the patient’s treatment regimen, potentially optimizing treatment response and prognosis. In the future, strategies aimed at ameliorating muscle loss and optimizing fat deposition, such as tailored resistance training and protein and amino acid supplementation, may play a pivotal role in enhancing the efficacy of immunotherapy and extending survival. Notably, reports suggest that L-carnitine supplementation could potentially improve skeletal muscle quality in patients with HCC undergoing treatment with lenvatinib (26). These avenues merit further exploration for their potential contribution to comprehensive and personalized cancer care.

The following several limitations are deemed noteworthy. Firstly, our study is inherently limited by its observational, retrospective, and non-randomized design, conducted exclusively at a single institution. However, a notable strength lies in our effort to enhance statistical reliability and mitigate selection bias through external validating using a separate cohort. Secondly, the heterogeneity of our study population arises from the varied immunotherapy regimens administered to our patients. Finally, patients who did not receive CT examinations or only MRI within 1 month before immunotherapy were not included in this study, which may have led to patient selection bias. Therefore, future prospective randomized and multicenter clinical trials accompanied by molecular investigations are warranted to advance the validating of these results, and to shed further light on the potential mechanisms role of changes in muscle and skeletal muscle fat in advanced HCC patients treated with ICIs.

In essence, these findings indicated that progressive sarcopenia and myosteatosis are predictors of poor clinical outcome in patients with advanced hepatocellular carcinoma receiving immune checkpoint inhibitors, and a high visceral adipose tissue index at baseline is correlated with worse overall survival.

## Data availability statement

The datasets presented in this article are not readily available due to restrictions on privacy and ethics. Requests to access the datasets should be directed to YL, liyunqiao@hust.edu.cn.

## Ethics statement

The studies involving humans were approved by Medical Ethics Committee of Union Hospital, Tongji Medical College, Huazhong University of Science and Technology. The studies were conducted in accordance with the local legislation and institutional requirements. The ethics committee/institutional review board waived the requirement of written informed consent for participation from the participants or the participants’ legal guardians/next of kin because This retrospective observational study was waived the requirement for informed consent as all data were analyzed retrospectively and anonymously.

## Author contributions

ML: Conceptualization, Data curation, Formal analysis, Investigation, Methodology, Software, Visualization, Writing – original draft. QJ: Conceptualization, Data curation, Investigation, Methodology, Project administration, Writing – review & editing. HW: Data curation, Investigation, Writing – original draft. YL: Conceptualization, Funding acquisition, Methodology, Project administration, Resources, Supervision, Validation, Writing – review & editing.
